# SUDEP risk is influenced by longevity genomics: a polygenic risk score study

**DOI:** 10.1016/j.ebiom.2025.105841

**Published:** 2025-07-28

**Authors:** Helena Martins, James D. Mills, Susanna Pagni, Medine I. Gulcebi, Angeliki Vakrinou, Patrick B. Moloney, Lisa M. Clayton, Ravishankara Bellampalli, Hannah Stamberger, Sarah Weckhuysen, Pasquale Striano, Federico Zara, Richard D. Bagnall, Rebekah V. Harris, Kate M. Lawrence, Lynette G. Sadleir, Douglas E. Crompton, Daniel Friedman, Juliana Laze, Ling Li, Samuel F. Berkovic, Christopher Semsarian, Ingrid E. Scheffer, Orrin Devinsky, Karoline Kuchenbaecker, Simona Balestrini, Sanjay M. Sisodiya

**Affiliations:** a University College London Queen Square Institute of Neurology, London, WC1N 3BG, UK; b Chalfont Centre for Epilepsy, Chalfont St Peter, SL9 0RJ, UK; c Department of (Neuro)Pathology, Amsterdam Neuroscience, Amsterdam UMC Location University of Amsterdam, Meibergdreef 9, Amsterdam, the Netherlands; d Department of Medical Pharmacology, Marmara University School of Medicine, Istanbul, Turkey; e Applied & Translational Neurogenomics Group, VIB Center for Molecular Neurology, VIB, Antwerp, Belgium; f Translational Neurosciences, Faculty of Medicine and Health Science, University of Antwerp, Antwerp, Belgium; g Department of Neurology, Antwerp University Hospital, Antwerp, Belgium; h IRCCS G. Gaslini, Pediatric Neurology and Muscular Diseases Unit, Full Member of ERN-EPICARE, Genova, Italy; i Department of Neurosciences, Rehabilitation, Ophthalmology, Genetics, Maternal and Child Health, University of Genova, Genova, Italy; j UOC Genetica Medica, IRCCS Istituto, Giannina Gaslini, Genoa, Italy; k Molecular Cardiology Group at Centenary Institute, The University of Sydney, Sydney, New South Wales, 2050, Australia; l Faculty of Medicine and Health, The University of Sydney, Sydney, New South Wales, 2050, Australia; m Epilepsy Research Centre, The University of Melbourne, Austin Health, Heidelberg, Victoria, 3084, Australia; n Department of Paediatrics and Child Health, University of Otago, Wellington, New Zealand; o Department of Neurology, Northern Health, Epping, Victoria, Australia; p Department of Neurology, NYU Grossman School of Medicine, New York, NY, 10016, United States; q Department of Pediatrics, University of Maryland School of Medicine, MD, 21223, United States; r Office of the Chief Medical Examiner (OCME), Baltimore, MD, 21201, United States; s Florey and Murdoch Children's Research Institutes, Victoria, 3052, Australia; t Department of Paediatrics, The University of Melbourne, Royal Children's Hospital, Parkville, Victoria, 3052, Australia; u University College London Division of Psychiatry, Maple House, London, W1T 7BN, UK; v Department of Neuroscience and Medical Genetics, Meyer Children's Hospital IRCSS, University of Florence, 50139, Florence, Italy

**Keywords:** Epilepsy, Death, Risk, Longevity, Intelligence

## Abstract

**Background:**

Sudden Unexpected Death in Epilepsy (SUDEP) is a rare and tragic outcome in epilepsy, identified by those with the condition as their most serious concern. Although several clinical factors are associated with elevated SUDEP risk, mechanisms underlying SUDEP are poorly understood, making individual risk prediction challenging, especially early in the disease course. We hypothesised that common genetic variation contributes to SUDEP risk.

**Methods:**

Genetic data from people who had succumbed to SUDEP was compared to data from people with epilepsy who had not succumbed to SUDEP and from healthy controls. Polygenic risk scores (PRSs) for longevity, intelligence and epilepsy were compared across cohorts. Reactome pathways and gene ontology terms implicated by the contributing single nucleotide polymorphisms (SNPs) were explored. In the subset of SUDEP cases with the necessary data available, a risk score was calculated using an existing risk prediction tool (SUDEP-3); the added value to this prediction of SNP-based genomic information was evaluated.

**Findings:**

Only European-ancestry participants were included. 161 SUDEP cases were compared to 768 cases with epilepsy and 1153 healthy controls. PRS for longevity was significantly reduced in SUDEP cases compared to disease (P = 0·0096) and healthy controls (P = 0·0016), as was PRS for intelligence (SUDEP cases compared to disease (P = 0·0073) and healthy controls (P = 0·00024)). The PRS for epilepsy did not differ between SUDEP cases and disease controls (P = 0·76). SNP-determined pathway and gene ontology analysis highlighted those related to inter-neuronal communication as amongst the most enriched in SUDEP. Addition of PRS for longevity and intelligence to SUDEP-3 scores improved risk prediction in a subset of cases (38) and controls (703), raising the area-under-the-curve in a receiver-operator characteristic from 0·699 using SUDEP-3 alone to 0·913 when PRSs were added.

**Interpretation:**

Common genetic variation contributes to SUDEP risk, offering new approaches to improve risk prediction and to understand underlying mechanisms.

**Funding:**

The Amelia Roberts Fund; 10.13039/100002736CURE Epilepsy; 10.13039/501100018726Epilepsy Society, UK; Finding A Cure for Epilepsy and Seizures (FACES).

**Research in context**

**Evidence before this study**

We searched PubMed from database inception to January 20, 2025, to identify papers published on SUDEP and common genetic variation without language restrictions, using the search terms “((SUDEP) AND (genetic) OR (genomic) AND (common) OR (SNP) OR (single nucleotide) OR (PRS)”. Only two publications with primary data were identified. One study examined 17 SNPs implicated in schizophrenia and epilepsy in a cohort of 340 cases of sudden cardiac death compared to 342 controls: an association was identified between the minor allele of the nonsynonymous SNP rs10503929 within the neuregulin 1 gene and sudden cardiac death. Another study identified combinations of SNPs and copy number variants in genes related to neurocardiac and respiratory control pathways in a single case of SUDEP in Dravet syndrome.

**Added value of this study**

To our knowledge, this study is the first to assess the contribution of common genetic variation to SUDEP risk. This common variation was addressed through polygenic risk score analyses using sets of common variants linked to traits of putative relevance to SUDEP, namely longevity and intelligence. Polygenic risk scores for both longevity and intelligence are reduced in individuals with epilepsy who had succumbed to SUDEP compared to surviving controls with epilepsy. The size of the SUDEP risk accounted for on the liability scale was modest: the longevity PRS explained ∼1% of the risk of SUDEP, whilst intelligence PRS explained ∼4% (R^2^ = 0·04) of the risk of SUDEP on a liability scale. Among our genotyped individuals, genetic information supplements the ability of clinical factors alone in predicting SUDEP risk.

**Implications of all the available evidence**

The precise risk of SUDEP in an individual patient remains difficult to predict. Our findings suggest that common genetic variation contributes to this risk. The literature also suggests that there may be an association between rare genetic variation and SUDEP, but more data are needed. Common and rare genetic variation can be determined at diagnosis by available tests, in contrast to most clinical risk factors that can only be measured some time after disease onset. Additional studies are required before polygenic risk scores can become part of clinical testing. Our results also open up new approaches to mechanistic understanding of SUDEP.

## Introduction

Sudden Unexpected Death in Epilepsy (SUDEP) is a tragic outcome in epilepsy, identified by the community affected by the condition as their most serious concern.^1^ SUDEP accounts for ∼1/1000 adult and ∼1/5000 childhood deaths annually among people with epilepsy, with higher rates in those with treatment-resistant epilepsy.^2^^,^^3^ Although agonal events leading to death have been documented from SUDEP occurring in Epilepsy Monitoring Units, the causes and pre-terminal mechanisms of SUDEP remain elusive.^4^ Some risk factors have been established: chronic epilepsy, particularly with frequent tonic-clonic seizures,^3^ seizures during sleep, male sex, and neurodevelopmental disability.^5^^,^^6^ A number of other potential associations, biomarkers and risk factors with lower evidential support include postictal generalised electroencephalography (EEG) suppression (PGES), reduced heart rate variability and pathogenic variants in genes related to respiratory control and cardiac arrhythmia.^3^^,^^7^^,^^8^ How these factors contribute to SUDEP risk remains unknown. Consequently, SUDEP is unpredictable at the individual level and challenging to prevent.^9^

No current method reliably predicts SUDEP. Risk scores, such as the SUDEP-CARE score, SUDEP-7, and SUDEP-3, have been developed from epidemiological studies.^10^^,^^11^ However, the need for further validation of these tools underscores their limitations and reflects gaps in understanding. Moreover, SUDEP can occur early in the disease course, perhaps even after the first seizure, or after extended periods of seizure remission, suggesting influences, such as innate factors, beyond any risk accumulated over the course of chronic epilepsy.

Within innate risk, genetic factors have garnered attention: pathogenic variants in single genes related to epilepsy or cardiac arrhythmia have been associated with an elevated risk of SUDEP,^8^^,^^12^^,^^13^ but by definition are rare. Common genetic variation plays an important role in some human traits, such as longevity and intelligence, and in diseases, including epilepsy.^14^, ^15^, ^16^, ^17^ Polygenic risk for disease can be as important as monogenic factors, and polygenic risk is of growing clinical interest.^15^^,^^18^

As SUDEP can usually be considered to represent premature mortality, examining the genetics of the counterpart of premature mortality, longevity,^16^ is one logical approach to identify innate, genetic, risk factors for SUDEP. Genetic factors associated with intelligence have also been associated with longevity, indicating the utility of studying these genetic risk factors. Further, longevity and intelligence have both been linked to multiple health outcomes, and may serve as a proxy for genomically-mediated resilience to health challenges.^16^^,^^17^ ApoE alleles are amongst the factors that feature in longevity genetics, and have been independently associated with health risks.^16^^,^^17^^,^^19^, ^20^, ^21^

We hypothesise that lower polygenic risk scores (PRS) for longevity and intelligence are associated with a higher SUDEP risk in individuals with epilepsy. We show that PRS for longevity is indeed lower in those who succumbed to SUDEP compared to a cohort of individuals with epilepsy who have not experienced SUDEP. Additionally, we found that the PRS for intelligence—but not for epilepsy—was lower in the SUDEP group. The findings may permit improved individual-level SUDEP risk prediction earlier in the disease trajectory, and start a process of identifying novel pathways of interest in SUDEP risk.

## Methods

### Ethics

This research was approved by the relevant ethics committees (UK: Camden & Kings Cross Research Ethics Committee, 11/LO/2016). For all cases from the UK and Europe, written informed consent for research use of clinical and genetic data was obtained from patients, their parents, or legal guardians in the case of those with intellectual disability during the life of the patient. For cases from Australasia, the human research ethics committees of Austin Health (Melbourne, Australia), Royal Prince Alfred Hospital (Sydney, Australia) and New Zealand approved the study. For patients recruited through the Epilepsy Research Centre, Melbourne and New Zealand, all patients, or their parents, next-of-kin, or legal guardian in the case of children or patients with intellectual disability, gave informed consent for epilepsy genetics research during the life of the patient. The senior next of kin gave further consent for ongoing research after SUDEP occurred. Informed consent was not required for the de-identified retrospective coronial SUDEP cases. For cases from the USA, blood samples were collected under two studies: Blood Spot Card study, which was exempt from the New York University (NYU) Institutional Review Board (IRB), and North American SUDEP registry (NASR) approved by NYU IRB.

### Cohort description

For all cohorts only individuals of European ancestry were considered. The ancestry was determined through comparison with the 1000 Genomes Project reference dataset.^22^ Principal component analysis (PCA) of the combined data was used to detect population structure down to the level of the reference dataset. A 2D PCA plot was used to visualise sample ancestry, and only samples from the cohorts which overlapped with the European-ancestry samples from the 1000 Genomes Project reference dataset were retained for further analysis (Supplementary Figs. S1, S2).

### SUDEP cohort

SUDEP cases were classified as definite SUDEP, definite SUDEP Plus, probable SUDEP, probable SUDEP Plus, or possible SUDEP,^23^ determined following discussion with the treating or recruiting epileptologist, review of the death certificates, available post-mortem documentation, and/or electronic medical records. Each SUDEP classification was defined as follows:^23^

• Definite SUDEP: Sudden, unexpected, witnessed or unwitnessed, nontraumatic and non-drowning death, occurring in benign circumstances, in an individual with epilepsy, with or without evidence for a seizure and excluding documented status epilepticus (seizure duration ≥30 min or seizures without recovery in between), in which postmortem examination does not reveal a cause of death.
• Definite SUDEP Plus: Satisfying the definition of Definite SUDEP, if a concomitant condition other than epilepsy is identified before or after death, if the death may have been due to the combined effect of both conditions, and if autopsy or direct observations/recordings of terminal event did not prove the concomitant condition to be the cause of death.
• Probable SUDEP/Probable SUDEP Plus: Same as Definite SUDEP but without autopsy. The victim should have died unexpectedly while in a reasonable state of health, during normal activities, and in benign circumstances, without a known structural cause of death.
• Possible SUDEP: A competing cause of death is present.
• Near-SUDEP/Near-SUDEP Plus: A patient with epilepsy survives resuscitation for more than 1 h after a cardiorespiratory arrest that has no structural cause identified after investigation.
• Not SUDEP: A clear cause of death is known
• Unclassified: Incomplete information available; not possible to classify

**Fig. 1:**
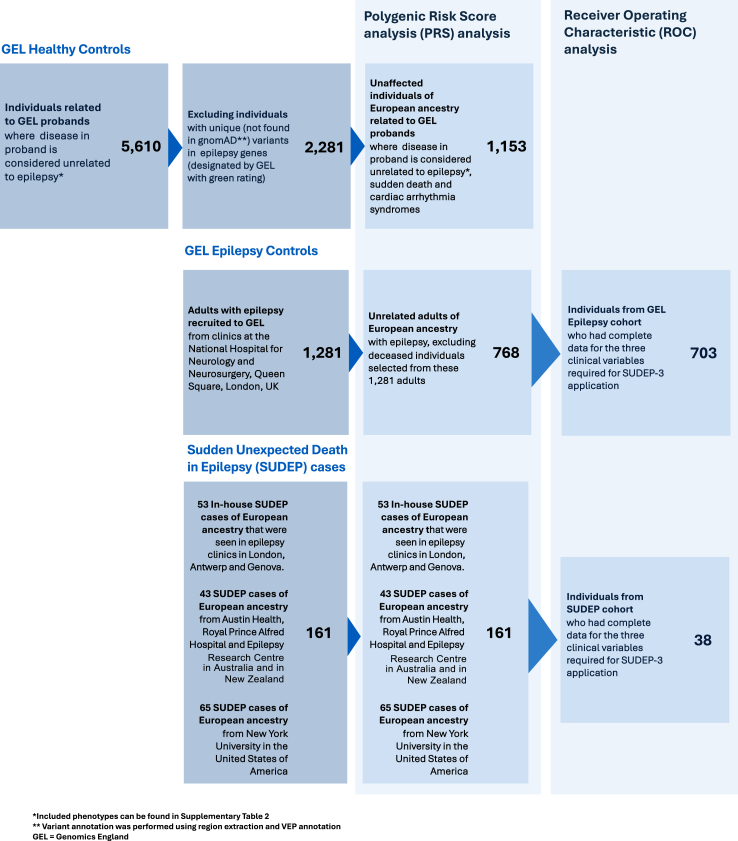
**Study design**. Description of cohorts used for polygenic risk score and ROC analysis.

Individuals classified as “definite”, “probable”, or “possible” SUDEP (including ‘Plus’ cases) (Supplementary Table S1) were included: all deceased between 2005 and 2024. Whole genome sequencing (WGS) data from three cohorts were combined: 53 cases that were seen in epilepsy clinics in London (n = 48), Antwerp (n = 3) and Genova (n = 2), and 108 cases from collaborators in Australia (n = 43) and USA (n = 65) (Fig. 1). Some individuals had donated DNA for genetic research years prior to death and may not have been under active follow-up at the time of death, precluding evaluation of some current known risk factors for SUDEP. Detailed demographic information of the SUDEP and control cohorts is available in Supplementary Table S2.

### Control cohorts

All control cohorts were compiled from participants recruited to the Genomics England (GEL) 100,000 genomes project.^22^

a. Epilepsy controls

The Epilepsy control cohort consisted of 768 adults with epilepsy recruited from clinics at the National Hospital for Neurology and Neurosurgery, London, UK, through the same approved study and clinics as the UK SUDEP cases (REC 11/LO/2016), and sequenced in the UK 100,000 genomes project (Fig. 1; Supplementary Table S2, ages given in Supplementary Table S3).^22^ All individuals fell within the GEL “epilepsy and other features” disease group.^24^ To minimise the possibility of inclusion of individuals within this cohort who might have succumbed to SUDEP, individuals deceased at the censor date were excluded; notably, the mean age at death of the SUDEP cohort was 35·9 years, and the mean age at last follow-up of the epilepsy cohort was 48·4 years. The Epilepsy cohort may include individuals who will ultimately succumb to SUDEP: any bias thus introduced would only lead to an underestimation in our analyses.

b. Healthy controls

The Healthy control cohort consisted of 1153 unaffected relatives of probands from GEL rare disease categories considered to be unrelated to epilepsy, sudden death and cardiac syndromes (Supplementary Table S4).^22^^,^^24^ Medical information regarding these individuals is unknown, and a proportion, likely reflective of the prevalence of active epilepsy in the UK, may have epilepsy, which would serve only to reduce the power of our comparisons. To minimise the number of individuals with potential “monogenic” epilepsies in the GEL Healthy control cohort who might potentially have a monogenic SUDEP risk factor, all individuals with unique variants (i.e. not present in the Genome Aggregation Database (gnomAD) in epilepsy-related genes in the GEL Genetic Epilepsy Syndromes (Version 4·1) panel were removed.^25^ Only genes designated by GEL with a “green” rating (i.e. those in which there is a high level of evidence for gene–disease association) were included and are referred to as “epilepsy-related genes”.^22^^,^^24^ The region of each epilepsy-related gene was extracted from variant call format and annotated using ANNOtate VARiation (ANNOVAR) (version 2019Oct24).

### Whole genome sequencing and data processing

#### Controls

WGS data of controls used in the analysis are extracted from aggregated 78,195 germline genomic VCFs (VCFs) from the 100,000 Genomes Project, which serves as a multi-sample VCF dataset (aggV2). All samples in the dataset were sequenced with 150bp paired-end reads in a single lane of an Illumina HiSeq X instrument and uniformly processed on the Illumina North Star Version 4 Whole Genome Sequencing Workflow (NSV4, v2·6·53·23), which comprises the iSAAC Aligner (v03·16·02·19) and Starling Small Variant Caller (v2·4·7). Samples were aligned to the *Homo sapiens* NCBI GRCh38 assembly with decoys. All samples included in aggV2 pass the following quality control filters: sample contamination (freemix) is less than 0·03, the ratio of single nucleotide variants (SNVs) heterozygous to homozygous calls is less than 3, and the total number of SNVs falls between 3·2 million and 4·7 million. Additionally, array concordance is greater than 90%, the median fragment size exceeds 250 base pairs, and the excess of chimeric reads is less than 5%. Furthermore, the percentage of mapped reads is greater than 60%, and the percentage of AT dropout is less than 10%.^26^

#### In-house SUDEP cases (from UK, Belgium, Italy)

For the in-house SUDEP cases (n = 53) WGS data was undertaken on DNA extracted from blood of the probands. Samples were prepared using the TruSeq DNA PCR-Free Library Kit (Illumina, San Diego, CA, USA) in accordance with the manufacturer's guidelines. All samples were subject to paired end sequencing of a read-length of 150 nucleotides to 30× coverage using a NovaSeq 6000 (Illumina, San Diego, CA, USA). Quality control of the Format for storing nucleotide sequences (FASTQ) files was performed using TrimGalore v0·6·3 (Babraham Institute, Babraham, Cambridgeshire, UK). All low-quality nucleotides and contaminating adaptor sequences were removed. Reads that were shorter than 100 nucleotides in length or lacking both forward and reverse orientations were excluded from the downstream analysis. Reads passing quality control were aligned to the human reference genome GRCh38 using Burrows-Wheeler Aligner v0·7·17,^27^ followed by marking of duplicate reads using Picard tools (v2·20·3) and Base Quality Score Recalibration. The resultant binary alignment map files were then processed through the genome analysis tool kit (GATK v4·1·2) according to the best practices pipeline for identification of variants and copy number variations.^28^

#### New York University

NYU provided raw WGS data from SUDEP cases, using DNA isolated from post-mortem blood samples. All samples were sequenced to a coverage of 2×, with paired-end reads of 150 nucleotides in length. Quality control of the FASTQ files was performed using TrimGalore v0·6·3 (Babraham Institute, Babraham, Cambridgeshire, UK). All low-quality nucleotides and contaminating adaptor sequences were removed. Reads that were shorter than 100 nucleotides in length or lacking both forward and reverse orientations were excluded from the downstream analysis. Reads passing quality control were aligned to the human reference genome GRCh38 using Burrows-Wheeler Aligner v0·7·17,^27^ followed by marking of duplicate reads using Picard tools (v2·20·3) and Base Quality Score Recalibration. The resultant binary alignment map files were then processed through GATK (v4·1·2) according to the best practices pipeline for identification of variants and copy number variations.^28^

#### University of Melbourne

For the SUDEP samples provided by the University of Melbourne, genomic DNA was isolated from post-mortem blood. Genomic DNA was isolated from postmortem blood using a Qiagen Mini Blood kit (Hilden, Germany). Genome sequencing was performed at the Australian Genome Research Facility, Victoria, Australia. TruSeq PCR free sequencing libraries were prepared according to the manufacturer's recommendations (Illumina, San Diego, CA) and paired-end with a read length of 150 nucleotides were performed on an Illumina NovaSeq X Plus platform. Sequencing reads were aligned to the human genome reference (GRCh38) using BWA-mem (v0·7·10). The resultant binary alignment map files were then processed through the GATK (v4·1·1) according to the best practices pipeline for identification of variants and copy number variations.^28^

### Polygenic risk scores

The PRS for longevity was calculated using published summary statistics from two meta-analyses of genome-wide association studies (GWAS) focussing on longevity, examining 11,262 and 3484 cases that survived to at least the age corresponding to the 90th and 99th survival percentile for their population background, respectively, as well as 25,483 controls who either died or were last contacted at an age corresponding to the 60th survival percentile.^16^ The GWAS for longevity has shown genome-wide significant single SNP associations for rs429358 variant (apolipoprotein E (ApoE) ε4, associated with decreased odds of surviving to the 90th and 99th percentile age), and rs7412 (ApoE ε2, associated with the opposite effect). Results shown for PRS for longevity include ApoE alleles in the PRS estimation. To test whether the ApoE alleles alone were driving the PRS findings, supplementary PRS analyses were conducted after excluding SNPs determining the ApoE allele (Supplementary Fig. S3).

As intellectual disability has been associated with SUDEP risk, we also evaluated a PRS for intelligence (using GWAS from Savage et al.^17^), hypothesising this would be lower in the SUDEP group compared to both control groups.^6^ PRS for intelligence was estimated, noting the absence of GWAS with available summary statistics for ‘intellectual disability’. The intelligence GWAS was derived from a meta-analysis of 269,867 individuals from 14 cohorts of European ancestry. Intelligence was assessed using various neurocognitive tests, primarily targeting fluid domains of cognitive functioning. Performance across the cognitive tasks were modelled as a latent factor denoted as g (the general factor of intelligence).^6^ To exclude the possibility that those who experienced SUDEP had a higher liability to epilepsy *per se*, PRS for epilepsy was also estimated in the SUDEP, Epilepsy and Healthy control cohorts, using the most recent International League Against Epilepsy GWAS summary statistics for epilepsy.^14^

To avoid over-interpretation of the PRS results, genetic correlation between intelligence, longevity, and epilepsy was estimated. The formal genetic correlation (*LD*-score) between the SNPs from GWAS summaries used for the PRS estimations was estimated.^14^^,^^16^^,^^17^ The genetic correlation coefficients (*rg*) was obtained using the Linkage Disequilibrium Score Regression (LDSC) tool.^29^ Intelligence and longevity are correlated (*rg* = 0·65), intelligence and epilepsy show a moderate negative genetic correlation (*rg* = −0·35), and epilepsy and longevity show relatively weak genetic correlation (*rg* = −0·16) (Supplementary Fig. S4). LDSC genetic correlation values are classified as strong when |rg| is ≥ 0·50, moderate when 0·30 ≤ |rg| < 0·50, weak when 0·10 ≤ |rg| < 0·30, and negligible when |rg| < 0·10. These values confirm a genetic correlation between the SNPs used to estimate PRS for Longevity and Intelligence. Therefore, from a conservative approach, as three PRS analyses were performed, the overall Adjusted P value significance threshold was set to *α* = 0·05/3 (0·017).^30^

A one-way ANOVA was applied to compare PRS between the three cohorts for the selected best-fit PT. The assumptions for ANOVA testing were considered. The three cohorts (SUDEP cases, GEL Epilepsy controls and GEL Healthy controls) were independent. Each cohort assessed for normal distributed using the Shapiro–Wilk normality test and the homogeneity of variances using the Bartlett test. For each test, all P values were >0·05.^31^ The analysis of the variance model was adjusted for sex and the first four principal components of ancestry. The first principal components (Principal Components (PC) 1 to 4) capture the greatest differences between samples (∼75% of cumulative variance in the study cohort) and are often associated with continental ancestry or major sub-populations. Including these components in the analysis adjusts for confounding effects caused by population stratification, thereby reducing biases in the interpretation of PRS.^32^

**Fig. 2:**
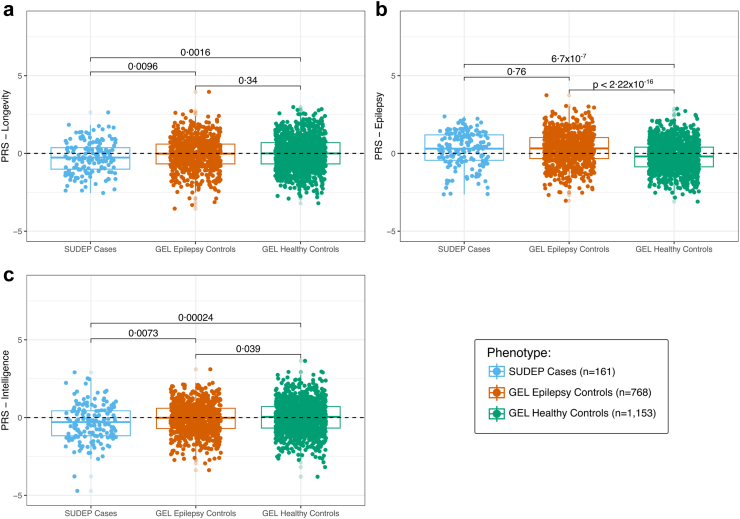
**Polygenic Risk Scores (PRS) applied across the cohorts**. (a) PRS for longevity was lower in the SUDEP cohort (n = 161) than in the Epilepsy (n = 768) (Adjusted P = 0·0096, at PT = 10^−3^, Tukey's test) and the Healthy control (n = 1153) cohorts (Adjusted P = 0·0016, at PT = 10^−3^, Tukey's test). The difference between the Epilepsy (n = 768) and the Healthy controls (n = 1153) was not significant (Adjusted P = 0·34, at PT = 10^−3^, Tukey's test). (b) PRS for epilepsy was not significantly different between the SUDEP cohort (n = 161) and the Epilepsy controls (n = 768) (Adjusted P = 0·76, at PT = 0·1, Tukey's test). PRS for epilepsy was significantly higher in the SUDEP cohort (n = 161) than in the Healthy controls (n = 1153) (Adjusted P = 6·6 × 10^−7^, at PT = 0·1, Tukey's test) and significantly higher in the Epilepsy controls (n = 768) compared to the Healthy controls (n = 1153) (Adjusted P < 2·22 × 10^−16^, at PT = 0·1, Tukey's test). (c) PRS for intelligence was significantly lower in the SUDEP cohort (n = 161) compared with the Epilepsy controls (n = 768) (Adjusted P = 0·0073, at PT = 0·01, Tukey's test) and the Healthy controls (n = 1153) (Adjusted P = 0·00024, at PT = 0·01, Tukey's test). PRS for epilepsy was not significantly different between the Epilepsy controls (n = 768) and the Healthy controls (n = 1153) (Adjusted P = 0·039, at PT = 0·01, Tukey's test). The per-PRS P values shown in the graphics are estimated using a post-hoc multiple pairwise comparisons (Tukey's test). As multiple PRS analyses were performed, the final Adjusted P value significance threshold was set to α = 0·05/3.

PRSs were estimated both with and without the application of Erase Sample Overlap and Relatedness (EraSOR).^33^ EraSOR helps to reduce inflation caused by sample overlap and close relatedness in the PRS. The results obtained before and after applying EraSOR were the same (see Fig. 2 and Supplementary Fig. S5).

### Polygenic risk scores: quality control steps

#### SNP quality control

Following the guidelines of Choi et al., quality checks were performed in the target and base data used for PRS estimation.^14^^,^^16^^,^^17^^,^^34^ PLINK 1·92 was used to remove all samples with <0·98 call rate for all single nucleotide polymorphisms (SNPs).^35^ Using a subset of uncorrelated SNPs (R^2^ < 0·1 in a sliding window of 100 SNPs per window and shifting the window by 25 SNPs each time), heterozygosity (HET), identity by state (IBS), represented by $\hat{\pi }$, and gender were calculated, and removed samples with: a) HET outliers >5 standard deviations from the median of the whole sample; ii) closely-related individuals from each identified pair, where $\hat{\pi }$ ≥ 0·125; iii) all samples where sex determined from genotype did not match with the reported sex. All SNPs with <0·95 genotype rate, <0·01 minor allele frequency, or deviation from Hardy–Weinberg equilibrium (with P < 1 × 10^−6^) in samples from any site, were also removed.

#### P value thresholding for polygenic risk score analysis

Following quality control steps, the PRS was calculated based on the overlap of the study groups’ remaining quality-controlled SNPs. PRS for each individual was obtained using the clumping and thresholding method implemented by Polygenic Risk Score software (PRSice) v2·3·5 across a set of P value thresholds (PT = 10^−4^, 10^−3^, 10^−2^, 5 × 10^−2^, 10^−1^, 0·5, 1). PT with the best fit for the target trait across the thresholds was identified (Supplementary Fig. S6–S12). R^2^ was used to measure the variance explained by the PRS and was produced directly from PRSice.

To identify the optimal PT for PRS prediction the software PRSice-v2·3·3 was used.^36^ This program permutes the target trait values across the sample of individuals 10000 times, and the PRS analysis is repeated on each set of permuted phenotypes. Thus, for each permutation, the “best-fit PRS” is obtained as that most associated (higher R^2^) with the target trait across the range of PTs considered.^36^ The PT with the most significant P value was chosen. The PRS for three phenotypes (intelligence, longevity and epilepsy) were estimated for three cohorts (SUDEP cases, GEL Epilepsy control, and GEL Healthy controls) as follows, assuming that the biological signal for common variant risk for all analysed phenotypes is the same irrespective of sample status. PRS was calculated using PRSice in a model that included the three cohorts setting GEL Healthy controls and GEL Epilepsy controls as controls, and SUDEP as cases. Using this model, the PT with the most significant P value was 10^−3^ for longevity PRS and 0·1 for epilepsy PRS and 10^−2^ for intelligence PRS (Supplementary Fig. S6).

To confirm that our approach did not force a single PT across the three groups, the PRS analysis was repeated three times applying PRSice in three different case vs control comparisons: SUDEP cases vs GEL Epilepsy controls, SUDEP cases vs GEL Healthy controls, and GEL Epilepsy controls vs GEL Healthy controls (Supplementary Figs. S7, S9 and S11). For all the three models, the PT with the most significant P value was 10^−3^ in the PRS for longevity (Supplementary Fig. S8), 0·1 in the PRS for epilepsy (Supplementary Fig. S10) and 10^−2^ in the PRS for intelligence (Supplementary Fig. S12). The results from this second approach are concordant with those of the first.^31^ The methods described above were also applied in the localised PRS analysis.

### ApoE status for individuals in the PRS analysis

The ApoE status of all individuals for whom PRSs were calculated was also determined. To assess the ApoE genotype of individuals in the PRS analysis, SUDEP cases, GEL Epilepsy control and GEL Healthy controls, the two SNPs located within the ApoE gene: rs429358 and rs7412 were extracted from VCF files. These SNPs are well-established markers for determining the ApoE alleles (ε2, ε3, and ε4), as they encode specific amino acid changes that impact the functionality of the ApoE protein and have been linked to a wide range of conditions, including cardiovascular diseases and neurodegenerative disorders, particularly Alzheimer's disease.^37^ The ApoE genotype is determined by the alleles present at these two SNPs as follows:

• rs429358 = C and rs7412 = C corresponds to the ε4 allele.
• rs429358 = T and rs7412 = C corresponds to the ε3 allele.
• rs429358 = T and rs7412 = T corresponds to the ε2 allele.

Based on the alleles from these SNPs, the ApoE genotype can be determined:

• ε2/ε2: Both SNPs are rs429358-T and rs7412-T.
• ε2/ε3: One SNP pair is rs429358-T, rs7412-T, and the other is rs429358-T, rs7412-C.
• ε3/ε4: One SNP pair is rs429358-C, rs7412-C, and the other is rs429358-T, rs7412-C.
• ε4/ε4: Both SNPs are rs429358-C and rs7412-C.

If an individual is heterozygous at both SNP locations (i.e. rs429358-C, rs7412-C and rs429358-T, rs7412-T), assigning a definitive genotype becomes challenging without phasing information. This configuration might suggest:

• ε2/ε4, where one chromosome carries rs429358-T, rs7412-T (ε2), and the other carries rs429358-C, rs7412-C (ε4).
• ε3/non-canonical, where one chromosome carries rs429358-T, rs7412-C (ε3), and the other carries rs429358-C, rs7412-T (non-canonical APOE allele).

Individuals who were heterozygous at both SNP locations (rs429358 and rs7412) were not included in the final ApoE genotype determination. Specifically, 20 (2·6%) samples from the GEL Epilepsy cohort, 26 (2·3%) from the GEL Healthy controls, and none from the SUDEP cohort were excluded due to this heterozygous configuration. This exclusion ensures that the ApoE genotypes reported are accurately phased and reflect either ε2, ε3, or ε4 alleles without ambiguity. The VCF files were analysed applying Bioinformatics tools for variant calling (bcftools) Version 1·9.^38^ Bcftools was used to extract the genetic variants located at the positions corresponding to rs429358 and rs7412, using genomic coordinates based on the reference genome, GRCh38. The extracted alleles for these SNPs were then examined to determine the ApoE allele in individuals used in the PRS estimations, excluding heterozygous configuration.

#### Pathways implicated by SNP set showing greatest effect size (based on PT)

For the longevity and intelligence PRSs, contributing SNPs were mapped to protein-coding genes (Supplementary Table S5) by positional mapping, identifying genes overlapping SNP coordinates or the nearest upstream or downstream genes based on genomic distance. Gene coordinates and annotations were obtained from Ensembl (GRCh38) using the BiomaRt R package, and genomic overlaps were identified using the GenomicRanges R package.^39^

Gene Ontology (GO) enrichment analysis and Reactome pathway analysis were conducted on gene sets using the clusterProfiler and ReactomePA R packages.^40^^,^^41^ P values were adjusted using the Benjamini-Hochberg method; enrichment results were considered significant with an adjusted P value threshold of <0·05.

### Evaluation of SUDEP-3 and polygenic risk scores as predictive classifiers for SUDEP risk

The SUDEP-3 score incorporates three clinical variables: presence of intellectual disability, any seizures in the past year, and >3 generalised tonic-clonic (GTC) seizures in the past year, to predict individual likelihood of SUDEP.^10^ Clinical data required to complete the SUDEP-3^10^ score were collected retrospectively by AV, LMC, PBM, MG and SB from electronic medical records and included:

1. History of a seizure of any type in the 12 months before the last follow-up or death
2. History of more than three tonic-clonic seizures in the 12 months before the last follow-up or death
3. A diagnosis of intellectual disability

Only a subset of individuals from the London, Antwerp, and Genoa cohorts had sufficient data for the SUDEP-3 calculation. The analysis included only individuals with clinical information pertaining to all three clinical components, which limited the Epilepsy control cohort to 703 individuals and the SUDEP cohort to 38 individuals.

The ability of the SUDEP-3 score, combined with longevity and intelligence PRSs, to distinguish individuals in the SUDEP cohort from those in the Epilepsy control cohort was evaluated using receiver operating characteristic (ROC) analysis using the R package pROC.^42^ The discriminative ability of each variable was first assessed individually (univariate analysis) and then in combination (multivariate analysis). Multivariable classifiers were constructed using generalised linear models (GLMs). The multivariable predictors included the combination of the intelligence and epilepsy PRSs; SUDEP-3 with the intelligence PRS; SUDEP-3 with the longevity RPS and SUDEP-3 with both the intelligence and epilepsy PRS. ROC curve analyses were performed for each model. DeLong's test was used to assess statistically significant differences between the ROC curves of each of the multivariable classifiers and the univariate SUDEP-3 classifier. The resulting P-values were corrected for multiple comparisons using the Bonferroni method. The DeLong test could not be applied to the intelligence or longevity PRS alone, as their inverse association with SUDEP risk led to score directions opposite to those of the multivariable classifiers, violating the test's assumptions.

### Statistics

Differences in the means between each pair of groups were evaluated for significance using a post-hoc multiple pairwise comparison with Tukey's test. The adjusted P values refer to those obtained following the application of Tukey's test in PRS analysis. Since multiple PRS analysis were conducted, Bonferroni correction was also implemented at the experiment-wide level, and the corrected experiment-wide significance level (α) was established at 0·05/n, where n represents the number of PRS analyses performed. For this study, with three PRS analyses conducted, the experiment-wide significance level was set to α = 0·05/3 (approximately 0·017).

### Role of funders

The study sponsors had no roles in study design; collection, analysis, and interpretation of data; writing of the report; or the decision to submit the paper for publication.

## Results

### PRS for longevity: common genetic variation contributes to SUDEP risk

PRS for longevity was significantly lower in the SUDEP cohort when compared to Epilepsy controls (Adjusted P = 0·0096, at PT = 10^−3^, Tukey's test) and to the Healthy controls (Adjusted P = 0·0016, at PT = 10^−3^, Tukey's test). Healthy controls and the Epilepsy controls were not different (Adjusted P = 0·34, at PT = 10^−3^, Tukey's test) (Fig. 2; Supplementary Figs. S7 and S8). The longevity PRS explained ∼1% (R^2^ = 0·01) of the risk of SUDEP on a liability scale (Supplementary Fig. S6a). Previous meta-analyses of GWAS for longevity identified the ApoE genotype as having a significant influence on lifespan.^16^ Accordingly, we assessed whether the ApoE genotype was driving the observed PRS results.^16^ In our analysis, individuals who were heterozygous at both SNP locations (rs429358 and rs7412) were not included in the final ApoE genotype determination as they do not allow unambiguous resolution of genotype. Only a minority of cases were affected (Table 1): specifically, 20 (2·6%) samples from the Epilepsy cohort, 26 (2·3%) from the Healthy controls, and no samples from the SUDEP cohort were excluded due to this heterozygous configuration (Supplementary Material 5). Across all cohorts, no significant differences (t-test) were observed between ApoE ε4 and ε2 genotypes (Table 1). Exclusion of ApoE SNPs from the PRS estimation did not alter the findings (Supplementary Fig. S3), showing that genetic factors beyond the ApoE genotype influence the longevity PRS results.

**Table 1:** Distribution of ApoE alleles in the study cohorts (SUDEP cases, Epilepsy and Healthy controls.

Genotype	Epilepsy controls (Total: 768)	Healthy controls (Total: 1153)	SUDEP cases (Total: 161)
ApoE ε3/ε4	189 (24·7%)	267 (23·1%)	38 (23·7%)
ApoE ε2/ε3	102 (13·3%)	136 (11·8%)	23 (14·3%)
ApoE ε4/ε4	18 (2·3%)	21 (1·8%)	3 (1·8%)
ApoE ε2/ε2	10 (1·3%)	6 (0·5%)	0 (0·0%)
ApoE ε3/ε3	429 (55·8%)	697 (60·5%)	97 (60·2%)

The sums for the controls do not add up to 100%, because individuals who were heterozygous at both SNP locations (ApoE ε2/ε4) were not included in the final ApoE genotype determination as they do not allow unambiguous resolution of genotype: 20 (2·6%) samples from the Epilepsy cohort, 26 (2·3%) from the Healthy cohort. No samples from the SUDEP cohort were excluded due to this heterozygous configuration.

### PRS for epilepsy: no difference between epilepsy control and SUDEP cohorts

The epilepsy PRS did not differ between SUDEP cases and Epilepsy controls (Adjusted P = 0·76, at PT = 10^−3^, Tukey's test). As expected, the epilepsy PRS was significantly higher in SUDEP cases compared with Healthy controls (Adjusted P = 6·6 × 10^−7^, at PT = 10^−3^, Tukey's test) and significantly higher in the Epilepsy cohort compared with Healthy controls (Adjusted P < 2·22 × 10^−16^, at PT = 10^−3^, Tukey's test) (Fig. 2; Supplementary Figs. S6b, S9, S10).

### PRS for intelligence: contribution to SUDEP risk

PRS for intelligence was significantly lower in the SUDEP cohort than in Epilepsy (Adjusted P = 0·0073, at PT = 10^−3^, Tukey's test), and Healthy controls (Adjusted P = 0·00024, at PT = 10^−3^, Tukey's test). The Epilepsy and Healthy controls did not differ (Adjusted P = 0·039, at PT = 10^−3^, Tukey's test) (Fig. 2; Supplementary Figs. S11 and S12). The intelligence PRS explained ∼4% (R^2^ = 0·04) of the risk of SUDEP on a liability scale (Supplementary Fig. S6c).

### Genetic correlation between longevity, epilepsy and intelligence

There is a degree of correlation between the three sources GWAS used to calculate PRS (Supplementary Fig. S4). However, the number of individual SNPs used in each PRS that overlap amongst those used in the PRS analyses is small (maximum ∼2% of total number of SNPs per PRS; Supplementary Fig. S13).

### Pathway analyses

No pathways were enriched as determined by SNPs from the longevity PRS analysis, whilst a number were enriched as determined by analysis of the SNPs from the intelligence PRS, focussed especially on inter-neuronal communication and glycosylation (Supplementary Fig. S14a). Only a few GO terms, mainly related to neuronal anatomy, are significantly enriched as determined by the longevity PRS, whilst for the intelligence PRS the terms again mainly relate to inter-neuronal connectivity.

### Augmenting the SUDEP-3 score with polygenic risk scores to improve predictive power

**Fig. 3:**
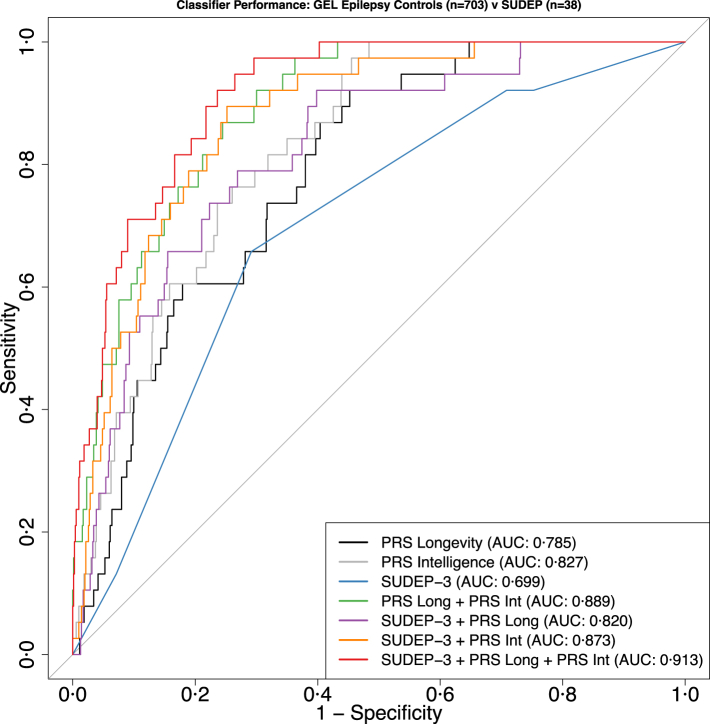
**Performance of different classifiers of SUDEP**. ROC analysis of all tested univariate and multivariate classifiers. PRS longevity (AUC: 0·785; 95% CI: 0·7251–0·8451), PRS intelligence (AUC: 0·827; 95% CI: 0·776–0·8776), SUDEP-3 (AUC: 0·699; 95% CI: 0·6221–0·7761), PRS longevity and PRS intelligence (AUC: 0·889; 95% CI: 0·8487–0·9287), SUDEP-3 and PRS longevity (AUC: 0·820; 95% CI: 0·7561–0·8828), SUDEP-3 and PRS intelligence (AUC: 0·873; 95% CI: 0·8254–0·9196), SUDEP-3 and PRS intelligence and PRS longevity (AUC: 0·913; 95% CI: 0·879–0·947).

When distinguishing individuals from the SUDEP cohort from the Epilepsy control cohort, the SUDEP-3 classifier achieved an Area Under the Curve (AUC) of 0·691 (95% Confidence Interval (CI): 0·6221–0·7761) (Table 2, Fig. 3). Subsequently, the PRS for longevity and intelligence were evaluated as univariate classifiers to distinguish individuals between the SUDEP cohort and the Epilepsy control cohort. Both the longevity PRS (AUC = 0·785; 95% CI: 0·7251–0·84519) and the intelligence PRS (AUC = 0·827; 95% CI: 0·776–0·8776), outperformed the discriminative ability of SUDEP-3 alone (Table 2, Fig. 3).

**Table 2:** Classifier performance.

Classifier	AUC (95% CI)	Specificity	Sensitivity	Adjusted DeLong's test P-value (Multivariable vs SUDEP-3)
PRS longevity	0·785 (0·7251–0·8451)	0·55	0·92	–
PRS Intelligence	0·827 (0·776–0·8776)	0·64	0·97	–
SUDEP-3	0·6991 (0·6221–0·7761)	0·71	0·66	–
PRS Longevity and PRS Intelligence	0·849 (0·8487–0·9287)	0·76	0·87	3·0 × 10^−4^
SUDEP-3 and PRS Longevity	0·820 (0·7561–0·8828)	0·60	0·92	1·2 × 10^−4^
SUDEP-3 and PRS Intelligence	0·873 (0·8254–0·9196)	0·75	0·89	4·81 × 10^−7^
SUDEP-3 and PRS Intelligence and PRS Longevity	0·913 (0·879–0·947)	0·76	0·92	5·82 × 10^−9^

Next, the discriminative ability of all possible combinations of the three variables (SUDEP-3, PRS for longevity, and PRS for intelligence) to distinguish SUDEP cases from Epilepsy control cases was evaluated. Augmenting SUDEP-3 with the PRS for longevity or the PRS for intelligence improved the discriminative performance of SUDEP-3 (Table 2, Fig. 3). Combining both the PRS for longevity and PRS for intelligence outperformed these classifiers (Table 2, Fig. 3). The best-performing classifier incorporated all three variables (SUDEP-3, PRS for longevity, PRS for intelligence), achieving an AUC of 0·913 (95% CI: 0·879–0·947) (Table 2, Fig. 3). Each of the multivariable classifiers demonstrated significantly greater discriminative performance than the SUDEP-3 classifier alone, as determined by DeLong's test (Adjusted P < 0·05, Table 2).

## Discussion

SUDEP has been identified as the top concern for people with epilepsy.^1^ A comprehensive understanding of the interacting proximate mechanisms and remote causes of SUDEP is crucial for developing effective prevention strategies. Proximate mechanisms are typically linked to seizures and their immediate pathophysiological effects, while remote causes may include genetic and environmental factors that shape the substrate on which proximate mechanisms act.^4^^,^^10^ Common genetic variation has not yet been studied systematically in the context of SUDEP.^13^ We show that common genetic variation, as measured by PRS, contributes to SUDEP risk: PRS for longevity and intelligence are both reduced in individuals with epilepsy who succumbed to SUDEP compared to those who have not. In contrast, PRS for epilepsy does not differ between these two groups. We showed that there is a degree of formal genetic correlation between the source GWAS, most notably between longevity and intelligence. However, the SNPs which contribute to the three PRSs show only a minor degree of overlap and given the pleiotropic effects of SNPs contributing to longevity and intelligence PRS especially, this minor overlapping of SNPs that generate the three PRS signals does not necessarily amount to redundancy of biological signal. We therefore present results for all three PRSs. We also included longevity and intelligence PRSs in the classifier analysis, which demonstrates, in the subset of cases for which data were available, that relevant genetic information can improve separation between cases and controls that was originally based on established clinical indicators alone. These findings have a number of implications.

Firstly, common genetic variation contributes to the risk of SUDEP. This observation is relevant to everyone with epilepsy, not only individuals who may have rare genetic variants causing their epilepsy. The finding opens up new vistas for further research into SUDEP risk and causation (including potential interaction between common and rare variant-mediated risk).^43^ Importantly, this genetic risk is present from birth and can be estimated in anyone with epilepsy at diagnosis, and may act in concert with risks that can only be determined later in the disease course (such as the occurrence of tonic-clonic seizures and the chronicity of epilepsy).

Secondly, the findings point to a possible better mechanistic understanding of SUDEP. The common variants contributing to risk are those related to longevity and intelligence. As a heuristic, the application of a PRS derived from the original longevity GWAS meta-analysis shows that people who succumbed to SUDEP were less likely *ab initio* to survive to the 90th or 99th age percentile than healthy controls or people with epilepsy who have not, by the time of the study, succumbed to SUDEP. Epilepsy may act as an ‘environmental’, or external, factor acting on this vulnerable genomic background to increase the risk of sudden death, in this case in the context of epilepsy and labelled ‘SUDEP’. Whilst there is established formal genetic correlation between longevity and intelligence (replicated here as expected), our pathway analyses, and the minimal overlap between SNPs contributing to the PRS signals, suggest the two PRSs may implicate non-duplicated biological signals and pathways, reflecting the pleiotropy of both measures.^16^^,^^17^^,^^44^ Notably, genetic contributions to both intelligence and longevity have been associated with a number of health outcomes, such as coronary artery disease, type 2 diabetes and father's age at death^16^ and BMI, waist-hip ratio, intracranial volume, schizophrenia, Alzheimer's disease, ADHD and autism and others (Supplementary Table S21 in ref.^17^): both can be viewed as collections of genetic factors with broad influences on health which have conveniently been visualised through the lenses of ‘longevity’ and ‘intelligence’, concepts which have served as portals to these sets of pleiotropic genetic influences. The *APOE* gene features in longevity genetics but not intelligence genetics^16^^,^^17^; it too has pleiotropic influences individually,^37^ though we show here that longevity genetics influences extend beyond *APOE* alone. Overall, further research is needed to determine how the PRS signals identified here may contribute to SUDEP risk, and whether those risks are mediated by known pleiotropic phenotypic associations of longevity and intelligence genetics, or through other means.

Thirdly, exploration of PRS in other sudden deaths, such as those labelled sudden cardiac death, or sudden death in infants, children or the young, may prove illuminating. Genetic associations with longevity^44^ and intelligence^17^ are pleiotropic, and considered to indicate resilience to health challenges, leading to suggestions for gerotherapeutics and lifestyle modifications. Whilst the pathway and gene ontology analyses must be considered preliminary, they point to multiple potentially contributing processes, especially those broadly focussed on inter-neuronal communication, of interest given the proposed roles for spreading depression and thalamocortical and brainstem autonomic network disruptions in SUDEP; as noted above, however, further research is needed to understand how the PRS signals identified might mediate risk for SUDEP.^45^ Importantly, the intelligence source GWAS found low enrichment for genes previously linked to intellectual disability,^17^ pointing to pathways beyond those involved in the clinical predictor ‘intellectual disability’, in keeping with the ROC findings. Moreover, the intelligence PRS does not indicate whether an individual is more or less likely to have intellectual disability.

Fourthly, incorporating PRSs for longevity and intelligence improved the performance of clinical operators (SUDEP-3)^10^ separating SUDEP cases from non-SUDEP epilepsy cases. Current SUDEP prediction tools continue to evolve and have had limited uptake in clinical practice, reflecting their limited performance in general.^10^^,^^11^^,^^46^ We show that incorporating innate risk due to common genetic variation as measured by PRS can improve performance, setting the stage for the development of better prediction tools, which may continue to improve as additional risk information accrues, for example from the combination of rare and common risk variants.^43^

There are limitations to our work. The number of individuals we were able to study who succumbed to SUDEP was small. Clinical (including syndromic), and investigational (eg PGES duration, heart rate variability), data on SUDEP risk was impossible to obtain from many of those studied due to the ascertainment and permissions processes. Three of 161 SUDEP cases included in PRS analyses were classified as ‘Possible SUDEP’, with a competing cause of death present. Overall, independent replication in larger, well-characterised cohorts is important, though will be challenging. We focussed on individuals of European ancestry: greater diversity of both SUDEP cases and source GWAS data is essential, and such cohorts are now accumulating.^14^ Information on potential rare variant contributions to SUDEP risk were not studied, and may further improve prediction tool performance, but this omission does not detract from the observed influence of common genetic variation. Further work is needed to determine how effective prediction algorithms incorporating different classes of genetic risk are, especially when combined with other investigation-based data—but an important feature of genetic risk factors is that they can be determined at diagnosis, do not need any duration of disease for their measurement and are cheaper and often more easily accessed, even in resource-poor settings, than many other investigations, such as EEG-videotelemetry.

In conclusion, common genetic variation contributes to SUDEP risk, and this observation opens up new fields of enquiry into this devastating outcome in epilepsy. The findings also do not exclude other polygenic trait contributions, and these, and other possible genetic contributions, also need to be explored in further studies. Although the clinical utility of PRS is still debated,^18^ this work raises new opportunities for the understanding, and, eventually, the prevention, of SUDEP.

## Contributors

HM and JDM were involved in conceptualisation, data curation, formal analysis, funding acquisition, investigation, methodology, project administration, visualisation, and writing—both the original draft and review & editing. SP contributed to formal analysis, investigation, methodology, visualisation, and writing—original draft and review & editing. MG, AV, PBM, and RB were involved in formal analysis, investigation, methodology, and writing—original draft and review & editing. LMC contributed to conceptualisation, funding acquisition, investigation, methodology, project administration, and writing—original draft and review & editing. HS, SW, PS, FZ, RDB, RVH, KML, LGS, DEC, DF, JL, LL, SFB, CS, IES, and OD contributed to writing—review & editing. KK was involved in formal analysis, methodology, and writing—review & editing. SB contributed to conceptualisation, funding acquisition, methodology, project administration, and writing—review & editing. SMS was involved in conceptualisation, formal analysis, funding acquisition, investigation, methodology, project administration, resources, supervision, and writing—original draft and review & editing.

All authors read and approved the final version of the manuscript. The authors HM, JDM, SP, MIG, AV, PBM, LMC, and RP accessed and verified the underlying data.

## Data sharing statement

Data used in this study originate from several sources, each with their own data use approvals and limitations. The study protocol is already specified in the article. Individual level deidentified participant genetic, summary clinical data and SUDEP classification data and a data dictionary defining each field in the set will be made available for non-competing analyses to bona fide researchers through the corresponding author subject to completion and approval of individual data sharing agreements related to each contributing source. Research on the de-identified patient data for the GEL Healthy Controls and GEL Epilepsy Controls used in this publication can be carried out in the Genomics England Research Environment subject to a collaborative agreement that adheres to patient led governance. All interested readers will be able to access the data in the same manner that the authors accessed the data. For more information about accessing the data, interested readers may contact research-network@genomicsengland.co.uk or access the relevant information on the Genomics England website: https://www.genomicsengland.co.uk/research.

No bespoke code was used for this study. All code used in the manuscript is in the public domain already and has been appropriately referenced.

## Declaration of interests

SFB was supported by NHMRC (grants 1091593; 1196637); received industry support from educational activities from UCB Pharma, Eisai, SEER, Chiesi and Liva Nova, received renumeration from Praxis Precision Medicines, Sequiris, Eisai, DeltaMed and the Epilepsy Foundation, and may accrues further revenue on a patent for SCN1A testing held by Bionomics Inc and licenced to various diagnostic companies. LGS receives consulting fess from the Epilepsy Study consortium. SW receives consultancy fees from UCB, Biocodex, Xenon Pharmaceuticals, Lundbeck, Knopp Biosciences, Encoded Therapeutics, Angelini Pharma, Liva Nova, Stoke Therapeutics and Roche. PS received consultancy fees from UCB, Biocodex, Desitin, Angelini Pharma and Jazz Healthcare. HS receives consultancy fees from Angelini Pharma, Biomarin, and Immedica. IES has served on scientific advisory boards for Bellberry Ltd, Chiesi, Eisai, Garvan Institue of Medical Research, Knopp Biosciences, Longboard Pharmaceutical, UCB, and Takeda Pharmaceuticals; has received speaker honoraria from Akumentis, BioMarin, Biocodex, Chiesi, Eisai, GlaxoSmithKline, Nutricia, Stoke Therapeutics, UCB, Zuellig Pharma; has received funding for travel from, Biomarin, Eisai, Stoke Therapeutics and UCB; has served as an investigator for Anavex Life Sciences, Biohaven Ltd., Bright Minds Biosciences, Cerebral Therapeutics, Cerecin Inc, Cerevel Therapeutics, Encoded Therapeutics, EpiMinder Inc, Epygenix, ES-Therapeutics, Longboard Pharmaceuticals, Marinus Pharmaceuticals, Neuren Pharmaceuticals, Neurocrine BioSciences, Ovid Therapeutics, Praxis Precision Medicines, Shanghai Zhimeng Biopharma, SK Life Science, Supernus Pharmaceuticals, Takeda Pharmaceuticals, UCB, Ultragenyx, Xenon Pharmaceuticals, Zogenix, and has consulted for Biohaven Pharmaceuticals, Care Beyond Diagnosis, Cerecin Inc, Eisa, Epilepsy Consortium, Longboard Pharmaceuticals, Praxis, Stoke Therapeutics, UCB and Zynerba Pharmaceuticals; and is a Non-Executive Director of Bellberry Ltd and a Director of the Australian Academy of Health and Medical Sciences and the Royal Society (Australia). She may accrue future revenue on the pending patents: WO/2006/133508: Patent for SCN1A testing held by Bionomics Inc and licenced to various diagnostic companies, WO/2013/059884: Patent for a molecular diagnostic/therapeutic target for benign familial infantile epilepsy (BFIE) [PRRT2] with royalties paid, WO2009/086591: May accrue future revenue on pending patent: Diagnostic And Therapeutic Methods For EFMR (Epilepsy And Mental Retardation Limited To Females). OD receives grant support from NINDS, NIMH, MURI, CDC and NSF. He has equity and/or compensation from the following companies: Tilray, Tevard Biosciences, Regel Biosciences, Script Biosciences, Actio Biosciences, Empatica, Ajna Biosciences, and California Cannabis Enterprises (CCE); he has received consulting fees or equity options from Emotiv, Ultragenyx, Praxis Precision Therapeutics; holds patents for the use of cannabidiol in treating neurological disorders but these are owned by GW Pharmaceuticals and he has waived any financial interests; holds other patents in molecular biology and is the managing partner of the PhiFund Ventures. DF receives salary support for consulting and clinical trial-related activities performed on behalf of The Epilepsy Study Consortium, a non-profit organization: he receives no personal income for these activities. NYU receives a fixed amount from the Epilepsy Study Consortium towards Dr. Friedman's salary. Within the past two years, the Epilepsy Study Consortium received payments for research services performed by Dr. Friedman from: Biogen, Biohaven, Cerberal Therapeutics, Cerevel, Encoded, Epalex, Equilibre, Jazz Pharmaceuticals, Jannsen, Longboard, Ludbeck, Marinus, Modulite, Neurocrine, Ono, Praxis, PureTech, Rapport Therapeutics, SK Lifescience, Supernus, UCB, and Xenon. DF has also served as a paid consultant for Neurelis Pharmaceuticals and Meili Technologies; has received travel support from the Epilepsy Foundation and research support from NINDS, NSF and CDC unrelated to this work. He holds equity interests in Neuroview Technology and has received royalty income from Oxford University Press. SMS has received honoraria for educational events or advisory boards from Jazz Pharma, Angelini Pharma, Biocodex, Eisai, Zogenix/UCB and institutional contributions for advisory boards, educational events or consultancy work from Eisai, Jazz/GW Pharma, Servier, Stoke Therapeutics, Takeda, UCB and Zogenix.
