# Supplementary material for: SUDEP risk is influenced by longevity genomics: a polygenic risk score study
Source: eBioMedicine. 2025 Jul 28;118:105841. doi: 10.1016/j.ebiom.2025.105841 (PMC12368338; doi:10.1016/j.ebiom.2025.105841)
Supplement: Supplementary Figures [file mmc1.docx]

​​**Supplementary Information:**

**1)** **Supplementary Figures**

**2)** **Supplementary Tables**

**Abbreviations:** AFR = African, AMR = American, EAS = East Asian, EPI = epilepsy, EUR = European, GEL = Genomics England, INT = intelligence, LONG = longevity PC1 = Principal Component 1, PC2 = Principal Component 2, PRS = Polygenic Risk Score, PT = P Value Threshold, SAS = South Asian, SUDEP = Sudden Unexpected Death in Epilepsy

1. **Supplementary Figures**

**
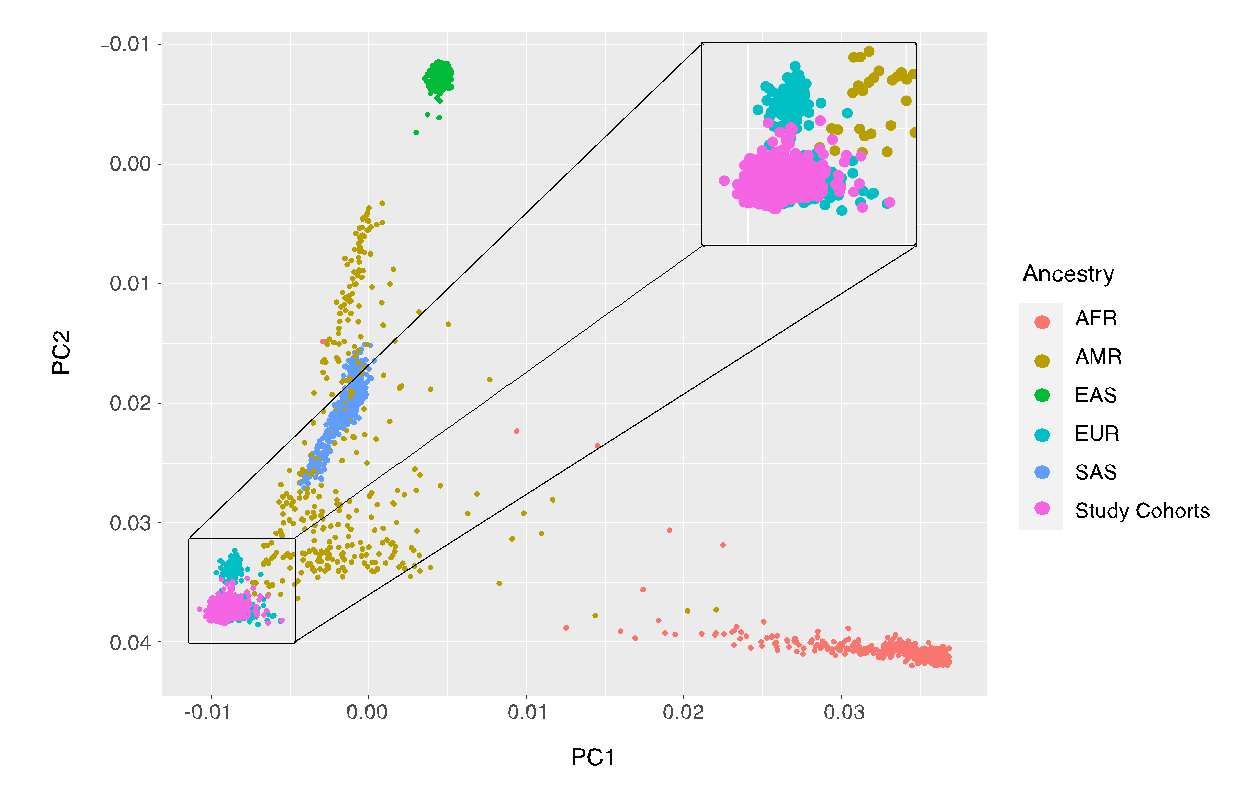
**

**Supplementary Figure 1:** Ancestry of the individuals in the study cohorts (Sudden Unexpected Death in Epilepsy (SUDEP) cases, Genomics England (GEL) Epilepsy controls, GEL Healthy controls) determined by comparison with the 1000 Genomes Project reference dataset. A principal component 1 (PC1) vs principal component 2 (PC2) plot was used to visualise sample ancestry. AFR: 1000 Genomes African samples; AMR: 1000 Genomes admixed American samples; EAS: 1000 Genomes East Asian samples; EUR: 1000 Genomes European samples; SAS: 1000 Genomes South Asian samples.

STUDY COHORTS: SUDEP Cases (n=161), GEL Epilepsy controls (n=768), GEL Healthy controls (n=1,153).


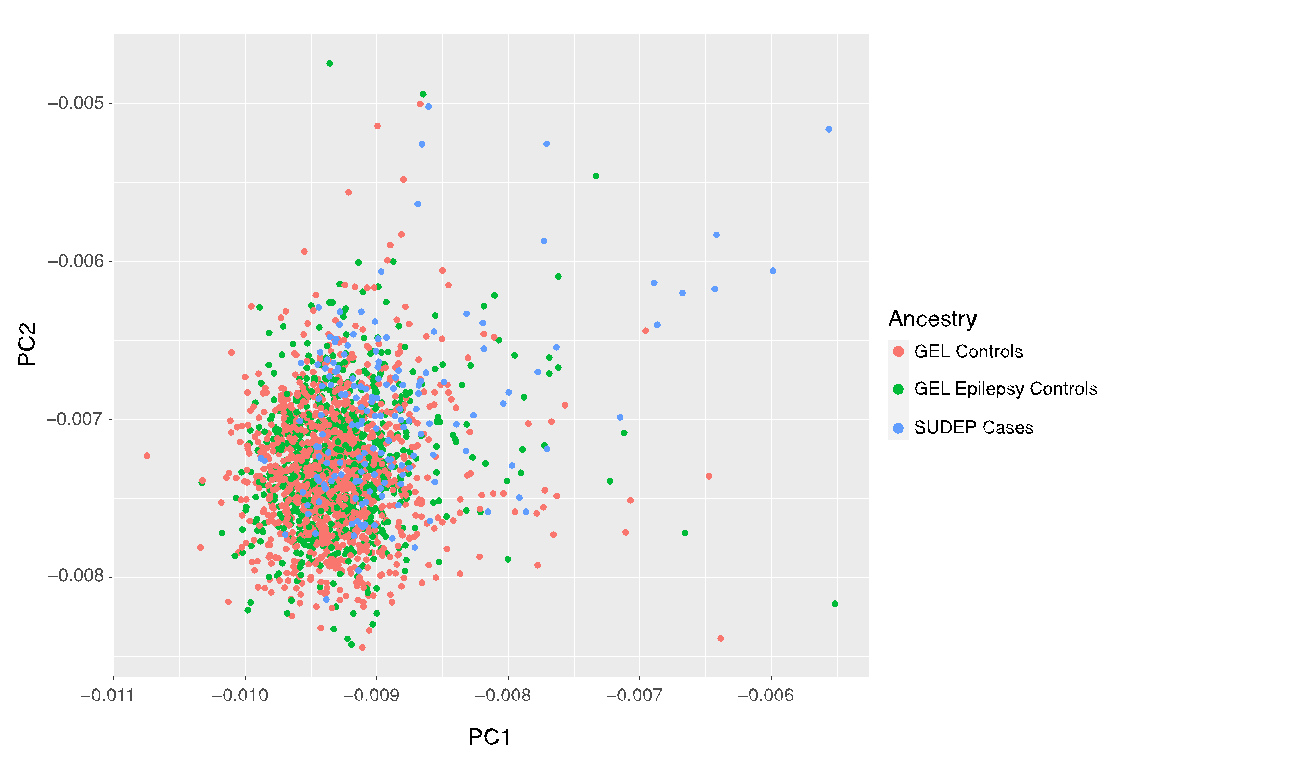


**Supplementary Figure 2:** Ancestry of the individuals in the SUDEP cases, GEL Epilepsy controls, GEL Healthy controls. A principal component 1 (PC1) vs principal component 2 (PC2) plot was used to visualise sample ancestry.

**
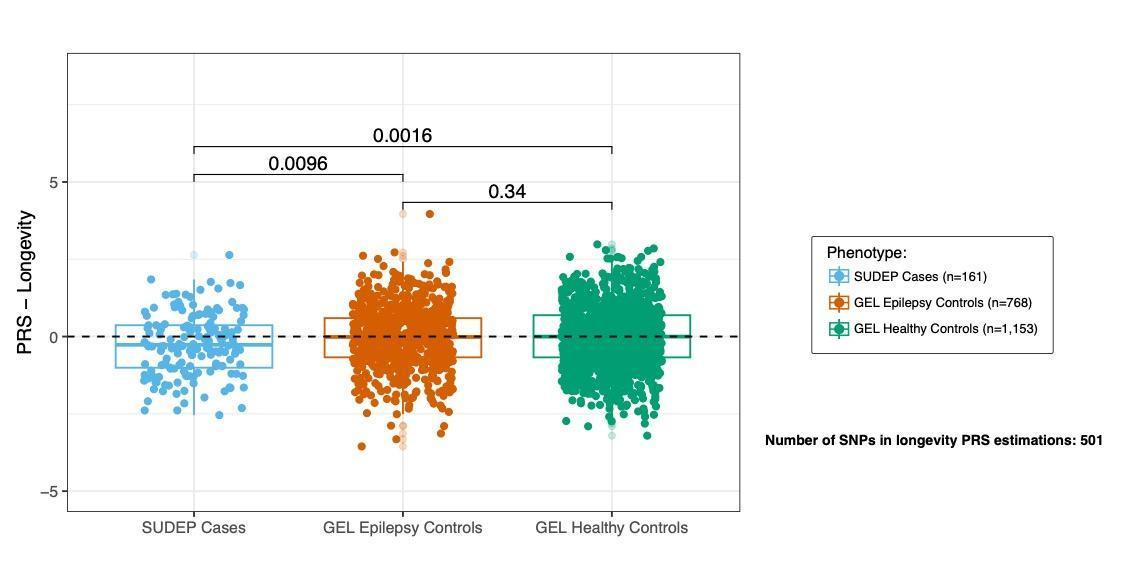
**

**Supplementary Figure 3:** Longevity Polygenic risk score (PRS) estimate without ApoE alleles. As for the primary analysis, PRS for longevity was lower in the SUDEP cohort than in the GEL Epilepsy (Adjusted P=0·0096, at PT=10^-3^, Tukey’s test) and the GEL Healthy control cohorts (Adjusted P=0·0016, at PT=10^-3^, Tukey’s test). The difference between the GEL Epilepsy and the GEL Healthy controls was not significant (Adjusted P=0·34, at PT=10^-3^, Tukey’s test).


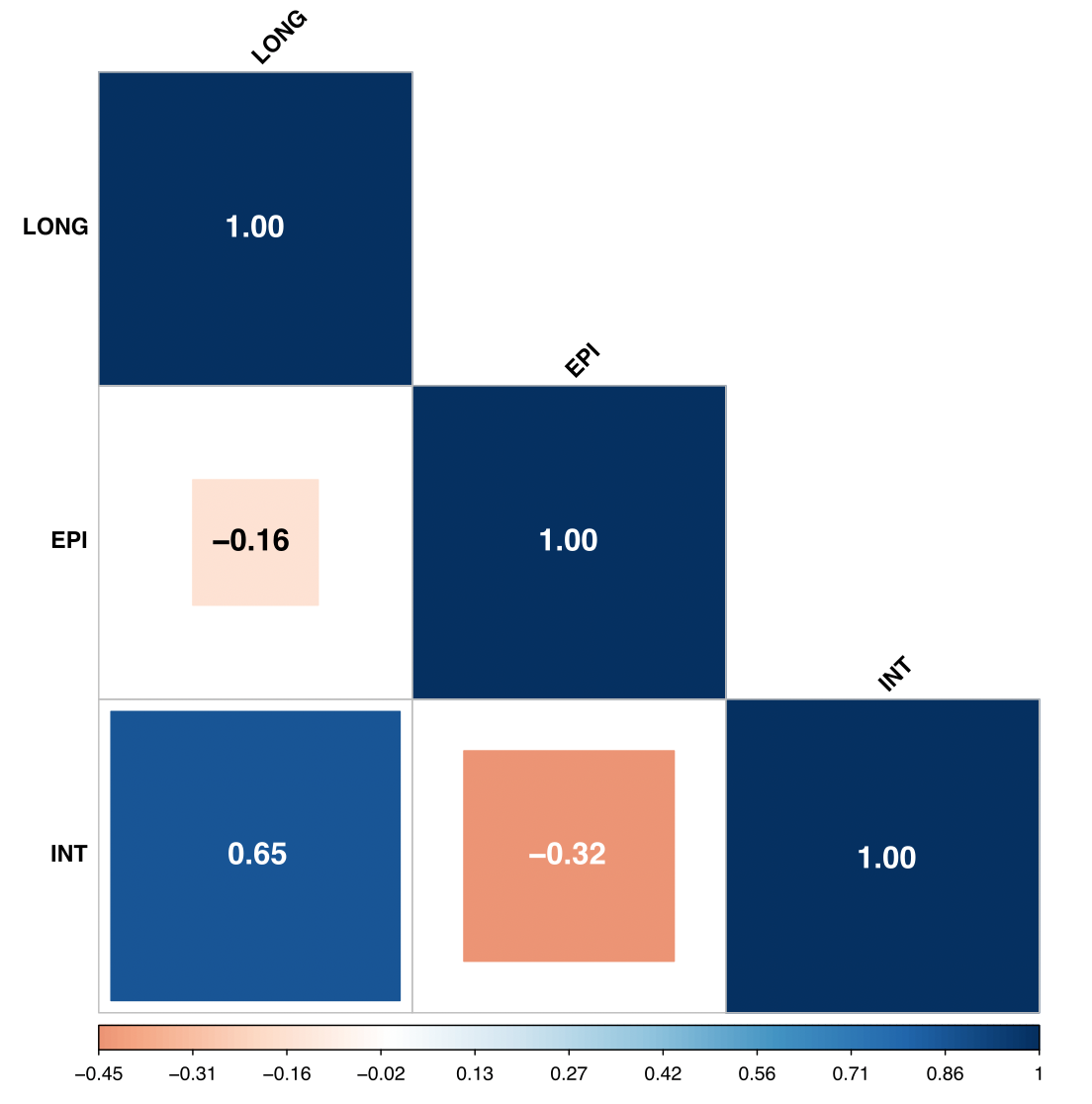


**Supplementary Figure 4**: **LD-score regression estimate between intelligence, longevity and epilepsy GWASs:** The genetic correlation coefficient (rg), calculated using LDSC tool^32^ is denoted with a colour scale ranging from -1 (red) to 1 (blue). Intelligence and longevity are genetically correlated (rg=0·65), intelligence and epilepsy show a moderate negative genetic correlation (rg=-0·32), and epilepsy and longevity show relatively weak negative genetic correlation (rg=-0·16). LDSC genetic correlation values are classified as strong when |rg| is ≥ 0·50, moderate when 0·30 ≤ |rg| < 0·50, weak when 0·10 ≤ |rg| < 0·30, and negligible when |rg| < 0·10. EPI = epilepsy; INT = intelligence; LONG = longevity.

**Supplementary Figure 5:** Polygenic Risk Scores (PRS) applied across the cohorts without use of the tool EraSOR.(a) PRS for longevity was lower in the SUDEP cohort than in the GEL Epilepsy (Adjusted P=0·0096, at PT=10^-3^, Tukey’s test) and the GEL Healthy control cohorts (Adjusted P=0·0016, at PT=10^-3^, Tukey’s test). The difference between the GEL Epilepsy and the GEL Healthy controls was not significant (Adjusted P=0·34, at PT=10^-3^, Tukey’s test). (b) PRS for epilepsy was not significantly different between the SUDEP cohort and the GEL Epilepsy controls (Adjusted P=0·76, at PT=0·1, Tukey’s test). PRS for epilepsy was significantly higher in the SUDEP cohort than in the GEL Healthy controls (Adjusted P=6·6x10^-7^, at PT=0·1, Tukey’s test) and significantly higher in the GEL Epilepsy controls compared to the GEL Healthy controls (Adjusted P<2·22x10^-16^, at PT=0·1, Tukey’s test). (c) PRS for intelligence was significantly lower in the SUDEP cohort compared with the GEL controls (Adjusted P=0·0073, at PT=10^-2^, Tukey’s test) and the GEL Epilepsy controls (Adjusted P=0·00024, at PT=10^-2^, Tukey’s test). PRS for epilepsy was not significantly different between the GEL Epilepsy controls and the GEL Healthy controls (Adjusted P=0·039, at PT=10^-2^, Tukey’s test). The per-PRS P-values shown in the graphics are estimated using a post-hoc multiple pairwise comparisons (Tukey’s test). As multiple PRS analyses were performed, the final Adjusted P value significance threshold was set to α=0·05/3.

**Supplementary Figure 6:** P value thresholding for polygenic risk score analysis: (a) Bar plot displaying the model fit of the longevity PRS at p value threshold (SUDEP cases vs GEL Epilepsy and GEL Healthy controls): The model fit of the longevity PRS shows a best predicting p value threshold at 10^-3^. The longevity PRS explained around 1% (R2=0·01) of the total phenotypic variance in the SUDEP cohort. 501 SNPs are used in the PRS estimation. (b) Bar plot displaying the model fit of the epilepsy PRS at p value threshold (SUDEP cases vs GEL Epilepsy and GEL Healthy controls): The model fit of the epilepsy PRS shows a best predicting p value threshold at 0·1. The epilepsy PRS explained around 0·8% (R2=0·008) of the total phenotypic variance in the SUDEP cohort. 16,166 SNPs are used in the PRS estimation. (c) Bar plot displaying the model fit of the intelligence PRS at p value threshold (SUDEP cases vs GEL Epilepsy and GEL Healthy controls): The model fit of the intelligence PRS shows the best predicting p value threshold at 10^-2^. To increase the number of SNPs involved in the estimation of PRS and, since the p value is significant for all p value thresholds, the PT equal to 10^-2^ was used in PRS for intelligence. The intelligence PRS explained around 4% (R2=0·04) of the total phenotypic variance in the SUDEP cohort. 9,254 SNPs are used in the PRS estimation.

**Supplementary Figure 7:** Longevity Polygenic Risk Score (PRS) for p value threshold equal to 10^-3^. (a) PRS for longevity estimation for SUDEP cases vs GEL Epilepsy controls: PRS for longevity was lower in SUDEP cohort than in GEL Epilepsy controls (Adjusted P=0·00036). (b) PRS for longevity estimation for SUDEP cases vs GEL Healthy controls: PRS for longevity was lower in the SUDEP cohort than in GEL Healthy controls (Adjusted P=0·0014), and (c) PRS for longevity estimation for GEL Epilepsy vs GEL Healthy controls cohorts: PRS for longevity was not significantly different between GEL Healthy controls and GEL Epilepsy controls (Adjusted P=0·12). The per-PRS p values shown in the graphics are estimated using a post-hoc multiple pairwise comparison (Tukey’s test). As three separate PRS analyses were performed, the adjusted p value significance threshold was set to α=0·05/3.

**Supplementary Figure 8:** Bar plot displaying the model fit of the longevity PRS at p value threshold in the models: (a) SUDEP cases vs GEL Epilepsy controls, (b) SUDEP cases vs GEL Healthy controls and (c) GEL Epilepsy vs GEL Healthy controls: The model fit of the longevity PRS shows the best predicting p value threshold at 10^-3^. 501 SNPs are used in the PRS estimation.

**Supplementary Figure 9:** Epilepsy Polygenic risk score (PRS) for p value threshold equal to 0·1. (a) PRS for epilepsy estimation for SUDEP cases vs Epilepsy controls: There was no significant difference in the epilepsy PRS between the SUDEP cases and GEL Epilepsy controls (Adjusted P=0·071). (b) PRS for epilepsy estimation for SUDEP cases vs GEL Healthy controls: PRS for epilepsy was significantly higher in the SUDEP cases than in GEL Healthy controls (Adjusted P=6·4x10^-7^). (c) PRS for epilepsy estimation for Epilepsy controls vs GEL Healthy controls: The epilepsy PRS was significantly higher in the GEL Epilepsy controls than the GEL Healthy controls (Adjusted P<2.22x10^-16^). The per-PRS p values shown in the graphics are estimated using a post-hoc multiple pairwise comparison (Tukey’s test). As thee separate PRS analyses were performed, the adjusted p value significance threshold was set to α=0·05/3.

**Supplementary Figure 10:** Bar plot displaying the model fit of the epilepsy PRS at p value threshold in the models: (a) SUDEP cases vs GEL Epilepsy controls, (b) SUDEP cases vs GEL Healthy controls and (c) GEL Epilepsy vs GEL Healthy controls: The model fit of the epilepsy PRS shows a best predicting p value threshold at 0·1. 16,166 SNPs are used in the PRS estimation.

**Supplementary Figure 11:** Intelligence Polygenic risk score (PRS) for p value threshold equal to 10^-2^. (a) PRS for intelligence estimation for SUDEP cases vs GEL Epilepsy controls: PRS for intelligence was lower in the SUDEP cohort than in GEL Epilepsy controls (Adjusted P=0·00045). (b) PRS for intelligence estimation for SUDEP cases vs GEL Healthy controls: PRS for intelligence was higher in GEL Healthy controls than in the SUDEP cohort (Adjusted P=0·00032), and (c) PRS for intelligence estimation for GEL Epilepsy controls vs GEL Healthy controls: There was no significant difference in intelligence PRS between GEL Healthy controls and GEL Epilepsy controls (Adjusted P=0·83). The per-PRS p values shown in the graphics are estimated using a post-hoc multiple pairwise comparison (Tukey’s test). As three separate PRS analyses were performed, the adjusted p value significance threshold was set to α=0·05/3.

**Supplementary Figure 12:** Bar plot displaying the model fit of the intelligence PRS at p value threshold: (a) SUDEP cohort vs GEL Epilepsy controls, (b) SUDEP cohort vs GEL Healthy controls and (c) GEL Epilepsy vs GEL Healthy controls: The model fit of the intelligence PRS shows a best predicting p value threshold at 10^-2^ in all study models. 9,254 SNPs are used in the PRS estimation.

**
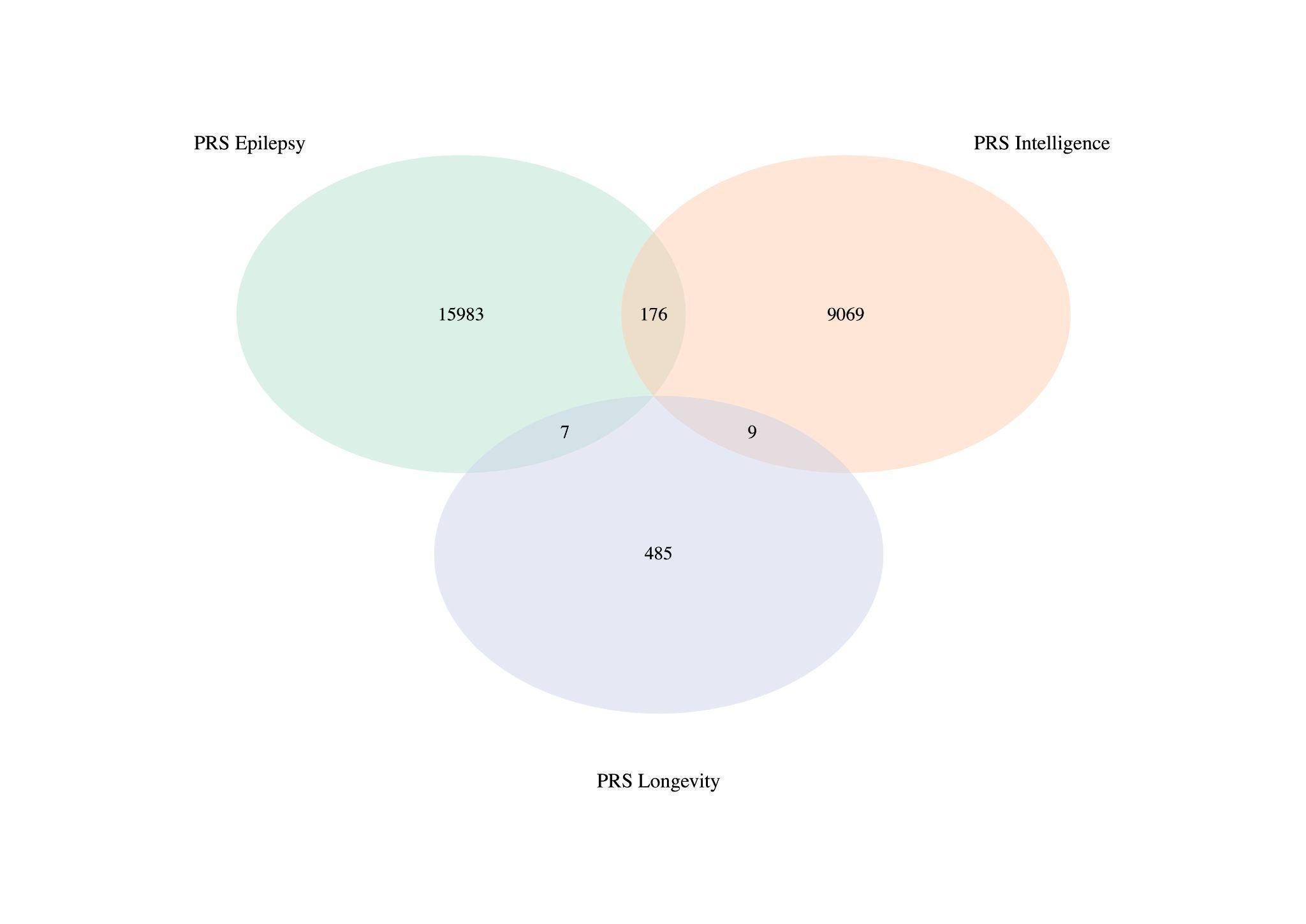
**

**Supplementary Figure 13:** Venn diagram showing the overlap of SNPs between the three PRSes. PRS Epilepsy is shown in green, PRS Intelligence in orange, and PRS Longevity is represented in blue. Each circle represents the set of SNPs associated with a specific PRS, while the overlapping regions indicate shared SNPs.

**Supplementary Figure 14:** (a) Bar plot showing the top 10 most statistically enriched Reactome pathways for the Intelligence PRS. The Longevity PRS did not yield any significantly enriched pathways. Each bar represents a Reactome pathway, with its length proportional to the number of genes mapped to that pathway. The colour scale ranges from red to blue, where red denotes pathways with higher enrichment significance (greater -log10 of the adjusted p-value), and blue represents lower significance. (b–d) Bar plots showing the top 10 most significantly enriched Gene Ontology (GO) terms for Biological Processes (b), Molecular Functions (c), and Cellular Components (d) across PRS analyses for Intelligence and Longevity. Bar lengths indicate the number of genes mapped to each GO term, with longer bars representing a higher gene count. Colours differentiate between PRS datasets: orange for Intelligence, and blue for Longevity. All displayed terms meet a significance threshold of an adjusted p value < 0·05.

**2. Supplementary Tables**

**Supplementary Table 1:** SUDEP classification and age at death of individuals in the SUDEP cases cohort.

**Supplementary Table 2:** Baseline demographic and clinical characteristics of individuals from the three study cohorts.

**Supplementary Table 3:** Age at last follow-up in the GEL Epilepsy cohort.

**Supplementary Table 4:** Genomics England disease categories considered unrelated to epilepsy.

**Supplementary Table 5:** List of genes to which SNPs from the longevity, intelligence and epilepsy PRSs were mapped.
